# Leptin as a key regulator of the adipose organ

**DOI:** 10.1007/s11154-021-09687-5

**Published:** 2021-09-14

**Authors:** Catalina Picó, Mariona Palou, Catalina Amadora Pomar, Ana María Rodríguez, Andreu Palou

**Affiliations:** Laboratory of Molecular Biology, Nutrition and Biotechnology (Nutrigenomics, Biomarkers and Risk Evaluation), University of the Balearic Islands. CIBER de Fisiopatología de La Obesidad Y Nutrición (CIBEROBN). Health Research Institute of the Balearic Islands (IdISBa), Palma, Spain

**Keywords:** Leptin, Adipose organ, Adiposity, Energy homeostasis, Metabolic programming, Development

## Abstract

Leptin is a hormone primarily produced by the adipose tissue in proportion to the size of fat stores, with a primary function in the control of lipid reserves. Besides adipose tissue, leptin is also produced by other tissues, such as the stomach, placenta, and mammary gland. Altogether, leptin exerts a broad spectrum of short, medium, and long-term regulatory actions at the central and peripheral levels, including metabolic programming effects that condition the proper development and function of the adipose organ, which are relevant for its main role in energy homeostasis. Comprehending how leptin regulates adipose tissue may provide important clues to understand the pathophysiology of obesity and related diseases, such as type 2 diabetes, as well as its prevention and treatment. This review focuses on the physiological and long-lasting regulatory effects of leptin on adipose tissue, the mechanisms and pathways involved, its main outcomes on whole-body physiological homeostasis, and its consequences on chronic diseases.

## Introduction: the discovery of leptin—a new perspective for the conception of adipose tissue and the study of obesity

The discovery of leptin in 1994 [1] represented a key milestone in the study of obesity and the knowledge of the molecular mechanisms involved in body weight control [2]. Leptin produced by the adipose tissue acts as an afferent signal in a negative feedback loop in the homeostatic control of adipose tissue mass, regulating food intake and energy expenditure [3]. Moreover, the finding that leptin was produced by the adipose tissue contributed to entail a change in the vision of this tissue. It is now considered as an endocrine organ, whose function goes much beyond that of mere storage of energy reserves, as it has implications in the maintenance of body homeostasis; this rekindled interest in this tissue [4]. In short, the discovery of leptin opened up a new molecular perspective in the conception of the adipose tissue and in the approach to the study and treatment of obesity, including new therapeutic targets, involving leptin itself, its regulation, and post-leptin processes downstream of leptin.

Leptin was initially described as an anti-obesity hormone. The lack of leptin in ob/ob mice was associated with early-onset, morbid obesity [1]. Leptin administration was found to restore normal body weight and reversed other alterations related to the absence of leptin, including hyperphagia, low body temperature, decreased metabolic rate, immunodeficiency, and insulin resistance [5–7]. Similar phenotypic abnormalities were found in humans with congenital leptin deficiency [8], and leptin administration was shown to effectively treat obesity, with the most significant impact being mediated by its suppressive effects on food intake [9]. But leptin protects not only against obesity, it really serves as an adiposity signal, with a critical function in maintaining adipose tissue mass to ensure survival in conditions of negative energy balance, thereby protecting individuals from the associated risks of having both a deficit and an excess of adiposity [3]. Nevertheless, human obesity is not generally caused by leptin deficiency, neither mutations of the leptin gene or the leptin receptor gene are frequent in humans, but leptin levels are commonly higher in subjects with obesity, and insensitivity to endogenous leptin is a hallmark of most cases of human obesity [10].

Although adipose tissue is the primary producer of leptin, other tissues also produce it, such as the stomach [11, 12]  - with a main function in the short-term regulation of food intake [13–15]  - as well as mammary gland [16] and placenta [17]  - with long-lasting effects in development [18]. Furthermore, we now know that leptin is involved in many other functions besides body weight control, including reproduction, glucose homeostasis, hematopoiesis, and immune function [19, 20]. Moreover, leptin plays essential roles during critical periods of development in foetal and infant development and as a potential programming factor [18], therefore exerting short, medium and long term actions, broadening the view of leptin as a pleiotropic hormone. For it, leptin exerts regulatory functions in different tissues, including the adipose tissue itself, and the action in this tissue represents a fundamental target in its main role in maintaining energy homeostasis. Comprehending the effects of leptin on adipose tissue and its regulation is critical to better understand the pathophysiology of obesity and related diseases, such as type 2 diabetes, as well as their prevention and treatment. Here, we aim to provide a comprehensive review of the main regulatory effects of leptin on adipose tissue under physiological and pathological conditions, such as obesity, the mechanisms and pathways involved, its influence in critical windows of development, and its long-lasting effects on metabolism and health.

## The endocrine adipose organ: leptin production and regulation

The adipose organ constitutes the main energy reservoir of the organism, which is the main place to accumulate the energy surplus as triglycerides (TG). This definition is undoubtedly an outdated view of the role of the adipose organ in metabolic homeostasis. Now, the pleiotropic functions of this organ are evident, among them being an endocrine organ that regulates metabolic pathways, which depends mainly on the type of cells and the location of the tissue [21, 22].

The adipose organ can be divided into two different main types of depots: white adipose tissue (WAT) depots, and brown adipose tissue (BAT) depots, with different functions and morphology [23]. Both types can be divided into various depots depending on their localization: subcutaneous, visceral, periaortic, etc., which exhibit different cellular composition, secretory capacity, vascularization, and innervation [24]. On the one hand, white adipocytes are unilocular cells, presenting one big fat vacuole that represents around 90% of the cell volume, where fatty acids (FA) are stored mainly as TG, which can be mobilized and distributed to different tissues when needed [23]. On the other hand, brown adipocytes are multilocular and rich in mitochondria, containing the uncoupling protein 1 (UCP1), a protein responsible for adaptive non-shivering thermogenesis, by uncoupling of the oxidative phosphorylation from ATP synthesis, exerting a key role on heat maintenance, particularly in newborns and hibernating mammals [25, 26]. Both types of cells are able to carry out the storage and mobilization of TG in response to the organism demands. Although the adipocyte is the characteristic cell, the adipose tissue also comprises other cell types such as stem cells, preadipocytes, fibroblasts, stromal cells, T-cells, granulocytes, macrophages and monocytes [24].

WAT displays a key role as an endocrine organ metabolically active by secreting a variety of functional products, called adipokines, which can act in autocrine, paracrine, or endocrine manners, establishing complex cross-talk between adipose tissue and other organs [24]. Probably, the so far considered most relevant adipokine is leptin, which is predominantly secreted from visceral fat in rodents [27] and from subcutaneous fat in humans [28]. The adipocytes secrete leptin into the bloodstream proportionally to fat mass; hence leptin levels are strongly correlated with adiposity in rodents and humans, serving as an adiposity signal [29].

Leptin expression and protein levels in the adipose tissue are regulated by many factors, including a variety of hormones, such as insulin [30], glucocorticoids [31, 32] and catecholamines [33], and show diurnal variations, according to the nutritional status [34]. In humans, leptin levels are high at night and low around noon and early afternoon period [35]. However, these changes are relatively modest (about 1.5-fold), compared to those associated with metabolic and nutritional states, which determine rapid changes in leptin synthesis regardless of changes in the size of fat depots [36]. Circulating leptin levels respond to nutritional state or feeding patterns, rising after feeding, when insulin levels are increased, and decreasing under fasting conditions or weight loss [29, 33, 37]. *In vitro* and *in vivo* studies support the direct role of insulin in leptin production. In starved rats, the combination of insulin administration and refeeding has been described to increase leptin mRNA expression to a similar extent to insulin alone, indicating that insulin is sufficient to mimic the effects of food intake on leptin expression [30]. Glucocorticoids also upregulate leptin mRNA expression, and chronic exposure to insulin further enhances leptin release [32]. In fact, the combination of hyperinsulinemia and the increased cortisol turnover that is associated with the obese state has been proposed to contribute to the maintenance of hyperleptinemia in obese individuals [38]. Conversely, the activation of the sympathetic nervous system via catecholamines contributes to a rapid decline in circulating leptin levels, therefore providing a mechanism in response to fasting and cold exposure [39, 40]. On the contrary, sympathetic blockade increases leptin gene expression and circulating leptin levels [41]. The fact that the sympathetic sensitivity of adipose tissue is reduced in obesity also contributes to explain the presence of increased leptin levels in the obese state, aside from the limited cases where leptin is absent [42].

The regulatory mechanisms involved in the transcriptional control of the leptin gene are poorly understood. Dallner et al. [43] have recently identified a fat-specific long non-coding RNA (lncOb), which is regulated in concert with fat mass and that regulates leptin expression by interacting with redundant enhancers. Lack of functional *IncOb* in mice is associated with decreased plasma leptin levels and increased adiposity, but these animals are responsive to leptin treatment. In humans, certain single-nucleotide polymorphisms in this locus are associated with decreased circulating leptin levels and obesity [43]. These findings seem clinically relevant regarding leptin therapy, particularly for the subset of patients with obesity who have relatively low circulating levels of leptin.

## Leptin action and leptin resistance

Leptin acts through its specific leptin receptor (LEPR), which exhibits a widespread distribution in the central nervous system (CNS) and peripheral organs. LEPR is encoded by the *LEPR* (or *OBR*) gene and belongs to the cytokine class 1 family [44]. Six isoforms have been identified, which have a common leptin-binding domain but differ in the length of C-terminal intracellular domain [45]. These isoforms include the long form (LEPRb) that contains the intracellular motifs required for full activation of the signalling pathways upon leptin binding, four short forms (LEPRa, LEPRc, LEPRd, and LEPRf), and one soluble form (LEPRe) without the transmembrane and cytoplasmic regions [46]. The reasons why distinct forms of leptin receptors are produced and their respective functions are not yet clear. The expression and characteristics of the leptin receptors isoforms, and the complete pathway of leptin signalling through LEPRb activation, can be revised elsewhere [46].

The principal neuronal targets of leptin are located in specific areas of the hypothalamus, a brain region with a key role in the control of feeding and energy expenditure [47]. The best-known leptin-sensitive neuronal systems start from the arcuate nucleus (ARC) of the hypothalamus, which contains neurons rich in the leptin receptor. Two main types of neurons with antagonistic functions are major targets of leptin. Leptin stimulates pro-opiomelanocortin (POMC) neurons responsible for the synthesis of POMC and cocaine‐ and amphetamine‐regulated transcript (CART), which are anorexigenic neuropeptides [48]. POMC is processed to form α-melanocyte-stimulating hormone (α-MSH), which is released from POMC axon terminals and activates melanocortin 3 and 4 receptors on downstream neurons [48]. Conversely, leptin acts on agouti-related peptide (AgRP) neurons in the ARC nucleus and inhibits the synthesis of the orexigenic AgRP and neuropeptide Y (NPY) neuropeptides, which may have complementary functions [49]. Both groups of neurons send projections to other hypothalamic nuclei involved in the control of energy balance, including the paraventricular nucleus (PVN) and the lateral hypothalamic area (LHA) [2]. Besides central action, leptin exerts a diverse range of peripheral actions related to its main roles in the regulation of energy balance [50, 51].

Leptin produced by the adipose tissue is not viewed primarily as a short-term satiety signal since circulating leptin levels may take several hours to change after food consumption [36]. However, leptin produced by the stomach may take part in the short-term control of food intake, together with other satiety signals [12, 15]. Leptin produced by the stomach is secreted to the gastric lumen and general circulation [12]. The amount of leptin released to circulation is probably not large enough to produce systemic effects as leptin produced by the adipose tissue does; rather, gastric leptin may act locally, and provide rapid information to the brain via peripheral vagal afferent pathways that originate in the stomach and intestine and terminate in the nucleus tractus solitarius, inducing satiety [15].

According to the anorexigenic role of leptin, exogenous leptin therapy reduces food intake and promotes weight loss in lean animals or humans with low circulating leptin levels [52–54]. In patients with congenital leptin deficiency, leptin is able to normalize energy balance, through a major effect on the reduction of food intake [55, 56]. Equally true is that most subjects with obesity are not deficient in leptin, rather exhibit higher circulating leptin levels than those in non-obese subjects, which is a feature of leptin resistance [10]. Subjects with generalized lipodystrophy and low leptin levels may also benefit from the administration of recombinant leptin as replacement therapy to manage metabolic diseases and comorbidities, since it has been reported to improve hypertriglyceridemia, glycaemic control, and overall liver health [57], and potentially reduce the risk of mortality [58].

Leptin resistance refers to the condition in which the brain or peripheral tissues are less sensitive (or do not respond) to leptin, and therefore leptin fails to promote its anticipated effects [59, 60]. This state could result in a vicious cycle, as it leads to a further increase in circulating leptin levels, and therefore to a worsening of leptin resistance. Therefore, leptin itself plays an important role in the development of its resistance [61]. The underlying mechanisms of leptin resistance are not fully clarified although, to date, several mechanisms have been proposed related to an impairment in leptin transport to the brain across the blood–brain barrier (BBB), or others alterations downstream of the leptin receptor, which are briefly discussed below (reviewed in [60]).

Leptin transport into the brain seems to be a limiting step in its central effects, but the way how blood leptin gains access to its target neurons in the brain is still an open question [60]. In subjects with obesity, elevated leptin levels in blood are not paired by proportionally high leptin levels in the cerebrospinal fluid, suggesting a causal relationship between a deficit in the transport system that carries leptin to the CNS and obesity [62]. Studies in obese mice show that these animals are sensitive to central administration of leptin but not to subcutaneous or intraperitoneal administration, indicating that the lack of leptin effect could be due to impaired transport of blood leptin to the brain [63]. However, the contribution of the alteration of leptin transport through the BBB to leptin resistance is not clear. Some studies have also shown that leptin resistant mice maintain intact transport of leptin through the BBB [64, 65], thereby its real contribution as a mechanism of leptin resistance has been questioned. Other studies in animal models have suggested that the impairment in the brain leptin transport is acquired rather than causal since it is developed secondary to obesity and may be reversible with weight reduction [66]. Therefore, although it is unlikely that alterations in the leptin transport through the BBB are directly involved in the development of leptin resistance, we cannot rule out that defects in this mechanism could play a role [20].

Of interest, recent studies have pointed out the role of tanycytes - a specialized glial cell type that line the third ventricle in the median eminence of the hypothalamus - in the shuttle of leptin and other hormones into the cerebrospinal fluid, also playing a potential role in the pathophysiology of central leptin resistance [67, 68]. In certain brain structures called the circumventricular organs, characterized by capillaries harbouring a fenestrated endothelium, the BBB consists of tanycytes, which extend from the wall of the cerebral ventricles and these fenestrated capillaries. These cells have barrier properties, preventing the diffusion of circulating molecules from fenestrated capillaries to the rest of the brain via the cerebrospinal fluid (see [68]). Tanycytes are possibly the first cells in the hypothalamus that respond to circulating leptin. They can take up leptin that freely exits the hypothalamic-pituitary portal blood circulation through fenestrated capillaries, and shuttle it into the brain to reach their target neuronal populations in an extracellular signal–regulated kinase (ERK)-dependent manner [67]. The transport of leptin seems to require the activation of a leptin receptor-epidermal growth factor receptor (LEPR–EGFR) complex [69]. Tanycytes appear to be the first cells of the brain to become resistant to leptin in diet-induced obesity, and this alteration is prior to the appearance of metabolic dysfunctions [68]. Interestingly, EGF treatment in diet-induced obese mice has been described to activate ERK in tanycytes, re-establish leptin transport, and restore the capacity of exogenous peripheral leptin to activate signal transducer and activator of transcription 3 (STAT3) in hypothalamic leptin-sensitive neurons [67].

Changes in the leptin receptor expression could also affect leptin sensitivity [70]. Hypothalamic *Lepr* mRNA (long form) downregulation has been found in animal models displaying hyperleptinaemia [71]. Interestingly, mechanisms leading to decreased *Lepr* mRNA expression may be early programmed [72]. Several studies have shown an association between the metabolic programming of obesity by adverse conditions during gestation and the presence of lower *Lepr* mRNA levels in the hypothalamus (often accompanied by a decrease in the expression of the insulin receptor), as well as an alteration in leptin signalling [73–76]. As an example, moderate calorie restriction during gestation in rats has been shown to be associated with higher food intake and higher body weight and adiposity in adulthood, in a sex-dependent manner, and this has been attributed, in part, to a diminished response to leptin action in adulthood [76, 77]. Notably, these animals already displayed lower *Lepr* mRNA levels in the hypothalamus at early stages of life (after weaning), suggesting central leptin resistance as an early adverse effect of gestational undernutrition, which may be responsible for later energy homeostasis dysregulation [76].

Overexpression of negative regulators of leptin action, such as the suppressor of cytokine signalling 3 (SOCS3) [78], and the tyrosine phosphatases (PTPs) tyrosine phosphatase 1 B (PTB1B) and T cell protein tyrosine phosphatase (TCPTP) [79], may also contribute to leptin resistance. Mice with haploinsufficiency of *Socs3* display greater leptin sensitivity than wild-type mice and are protected against the development of diet-induced obesity [80]. In turn, hypothalamic expression of PTPs has been found to be increased in obese animals, and selective ablation of these proteins partially prevents diet-induced obesity and improves leptin sensitivity [81–83], providing a demonstration of their role in the onset of leptin resistance. Moreover, and possibly in connection with the key role of SOCS3 and PTP1B in leptin action, endoplasmic reticulum (ER) stress has also been identified as a mechanism implicated in the development of obesity-associated leptin resistance [60, 84, 85]. ER stress may be the result of an excessive accumulation of unfolded proteins, activating the unfolded protein response (UPR) [86]. There is evidence that leptin resistance may be mediated, at least in part, by SOCS3 and PTP1B [60]. The expression of these proteins is inhibited by X-box binding protein 1 (XBP1), a main regulator of ER folding capacity [60]. Pharmacological induction of ER stress or deletion of XBP1 in neurons result in hyperleptinemia and obesity, associated with severe hypothalamic leptin resistance [84]. Conversely, selective overexpression of XBP1 in POMC neurons prevents the ER stress-mediated blockade of LEPRb and restores leptin sensitivity [84, 87].

## Regulatory actions of leptin in the adipose organ

As explained above, leptin exerts central actions, modulating energy homeostasis and influencing multiple actions on different tissues. The adipose tissue itself is an important target of leptin action, via autocrine, paracrine or endocrine signalling, fundamental for its main role in energy homeostasis [36, 88]. This action can differ depending on the depot and type of adipocytes, white or brown, and includes, besides the control of the main lipid metabolism pathways, other physiological processes such as adipogenesis, apoptosis, thermogenesis and browning, and inflammation [88]. Its knowledge is important for understanding the pathogenesis and pathophysiology of obesity and type 2 diabetes.

### Lipid metabolism

**Fig. 1:**
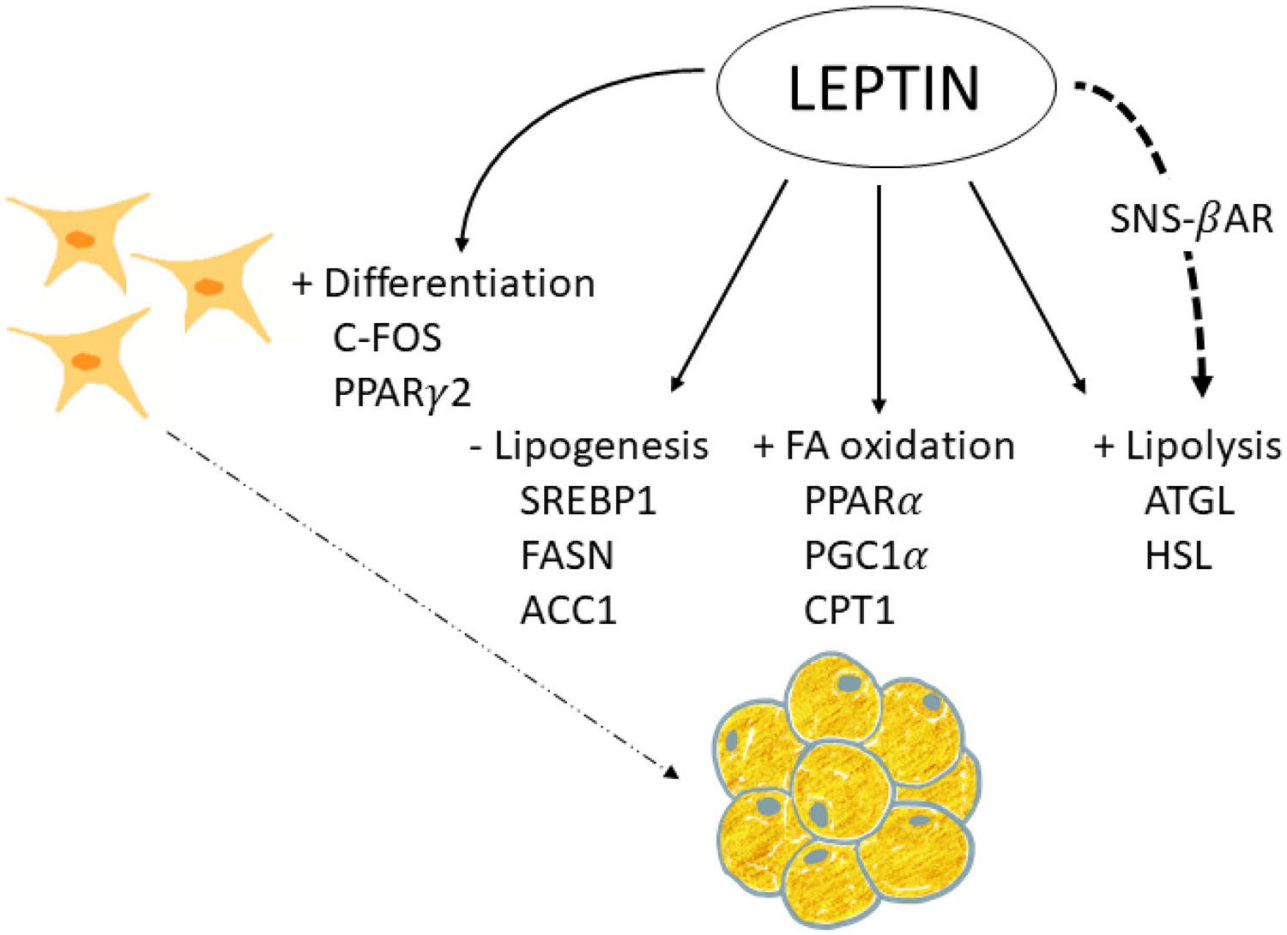
Leptin action on lipid metabolism and adipogenesis. Leptin is able to activate lipolysis in white adipose tissue (WAT), by the increase of ATGL (adipose triglyceride lipase) and HSL (hormone sensitive lipase) expression, both acting centrally activating sympathetic nervous system (SNS) and by a direct action on WAT, through β-adrenergic receptors (β-AR). Moreover, leptin directly stimulates fatty acid (FA) oxidation, by the increase of the expression of the genes coding for PPARα (peroxisome proliferator-activated receptor alpha), PGC1α (peroxisome proliferator-activated receptor-gamma coactivator 1alpha) and CPT1 (Carnitine palmitoyltransferase 1), among other proteins, and downregulates lipogenesis, decreasing the expression of SREBP1 (sterol regulatory element-binding protein 1), FASN (fatty acid synthase), ACC1 (acetyl-CoA carboxylase), etc., in WAT. In addition, leptin has been shown to exert direct actions stimulating pre-adipocyte differentiation, mainly through the activation of PPARγ2 (peroxisome proliferator-activated receptor gamma 2) and C-FOS, into mature adipocytes

Proper regulation of lipid metabolism is essential to prevent the development of obesity and metabolic diseases. Leptin is involved in regulating lipid metabolism in the adipose organ (Fig. 1), both directly by its interaction with its receptor [89, 90] and indirectly via sympathetic innervation [91]. However, the direct effects of leptin on the adipose tissues seem to be modest compared with the actions through the SNS [36].

The lipolytic effect of leptin on the adipose tissue is mainly mediated through sympathetic nerve fibres, establishing neuro-adipose junctions [91]. The effects of leptin involve the release of catecholamines and activation of β-adrenergic receptors (βAR), probably the β3AR, together with other potential stimuli [91, 92]. Surgical denervation of WAT prevents the effects of leptin activating hormone sensitive lipase (HSL), suggesting that sympathetic innervation of the tissue is required for the lipolysis-stimulating effects [91].

Besides the main effects of leptin through noradrenergic signalling, there is evidence from *in vitro* studies of a direct action of leptin on lipid metabolism in cultured/isolated white adipocytes, down-regulating lipogenesis and up-regulating lipolysis and FA oxidation, without the participation of the CNS [89, 90]. Concretely, leptin inhibits insulin-stimulated *de novo* FA synthesis [89], and this effect is produced by decreasing the expression of the enzyme acetyl CoA Carboxylase (ACC), which catalyses the first rate-limiting step in FA biosynthesis [93], and of fatty acid synthase (FAS), the enzyme responsible for *de novo* FA synthesis [94]. In addition, leptin increases the synthesis of nitric oxide, reducing glycerol synthesis, which reduces FA esterification [95].

Leptin-induced lipolysis has been demonstrated in adipocytes from lean and ob/ob mice, but not adipocytes from db/db mice that lack functional leptin receptor [96]. The direct lipolytic action of leptin is triggered by stimulation of triglyceride lipase (ATGL), the principal lipase at the initial step of TG hydrolysis, with no apparent effects on perilipin or HSL [90]. Besides lipolysis, leptin stimulates FA oxidation *in vitro*, increasing mRNA levels of the key transcription factor peroxisome proliferator-activated receptor alpha (PPARα) [94], the transcriptional coactivator persoxisome proliferator-activated receptor gamma coactivator 1alpha (PGC1α) [97], and its regulated genes coding for carnitine palmitoyl transferase-1 (CPT1) and acyl CoA oxidase (ACO) [94]. Leptin also increases the activity of citrate synthase and of the citric acid cycle [98, 99], contributing to the increase in FA oxidation.

It seems paradoxical that leptin increases lipolysis in adipose tissue when the circulating concentration of this hormone decreases under fasting conditions [36]. Furthermore, considering that the concentrations of leptin that have been shown to be effective in stimulating lipolysis *in vitro* are supraphysiological doses [90, 100], it seems unlikely that leptin could play a significant role in energy mobilization under energy deficit conditions. Instead, leptin could do this direct action under conditions of positive energy balance [90]. Likewise, it should also be considered that intra-adipose tissue leptin concentration could be higher than that present in circulation; therefore its effects through the autocrine or paracrine route could be higher, as has been suggested [90]. It has been proposed that the effects of leptin on lipolysis could depend on additional factors, enhancing or mediating its effects *in vivo*, such as circulating insulin levels and sympathetic activity, adjusting in a more precise way the use of fuels under specific metabolic conditions [36].

Findings from *in vivo* studies in rodents, in which leptin was administered peripherally, or with adenovirus-induced hyperleptinemia, support the role of leptin on adipose tissue metabolism, increasing lipolysis and decreasing fat synthesis, independently of its effects on food intake [101–103]. Subcutaneous leptin administration was associated with a progressive reduction in body weight and adiposity in different WAT depots (subcutaneous, intra-abdominal, and retroperitoneal) and in interscapular BAT, and this effect was associated with an increase in mRNA levels of lipoprotein lipase (LPL) and HSL, along with a decrease in mRNA levels of FASN, cytochrome c oxidase and leptin in the different fat depots [101]. A microarray performed in WAT from mice that were subcutaneously treated with leptin revealed a down-regulation of the lipogenic transcription factor Sterol regulatory element binding protein 1 (SREBP1), together with a cluster of lipogenesis-related genes regulated by SREBP1 [102]. Lean rats with adenovirus-induced hyperleptinemia also underwent a reduction in their adiposity, associated with both the inhibition of the expression of the lipogenesis-related genes coding for ACC, FASN and glycerol phosphate acyltransferase (GPAT) and their transcription factor, PPARγ, in epididymal WAT, and the stimulation of the expression of FA oxidation-related genes, coding for CPT1 and ACO, and their transcription factor PPARα [103].

### Adipogenesis

Adipogenesis is the process of differentiation of preadipocytes to adipocytes, which is dependent on the energy status and is controlled by hormone activity and at the transcriptional level [104]. This process allows adipose tissue to grow namely in response to an insufficient lipid storage capacity, thereby preventing ectopic fat deposition. Besides increasing lipolysis and FA oxidation and decreasing insulin-stimulated lipogenesis, leptin may affect the size of adipose tissue depots through its action on adipogenesis [105] (Fig. 1).

*In vitro*, leptin has been shown to have proadipogenic effects at physiological concentrations (10 nM), increasing proliferation and differentiation of primary cultured subcutaneous preadipocytes from rats [105]. This was associated with increased expression of differentiation markers (LPL and glycerol-3-phosphate dehydrogenase -GPDH) and two key adipogenic transcriptional factors C-FOS and PPARγ2, together with increased fat storage in these cells [105]. Leptin treatment at concentrations of 8 or 80 ng/mL had similar effects on the murine lineage of preadipocytes 3T3-L1 and in primary mouse adipose tissue-derived stromal cells increasing the expression of the adipogenesis- and lipogenesis-related proteins PPARγ, SREBP1C, Perilipin 1 (PLIN1), and caveolin 1 (CAV-1) [106]. Other studies have also shown that a relatively low concentration of leptin (50 ng/ml) stimulates proliferation of preadipocytes from rats in primary culture, whereas leptin at higher concentrations (250 and 500 ng/ml) inhibits proliferation of both preadipocytes and stromal vascular cells [107]. However, there are controversial results, since a high dose of leptin of 1000 ng/ml has been shown to stimulate the proliferation of porcine preadipocytes [108].

Therefore, the action of leptin on adipogenesis is complex, and its effects *in vivo* must be considered according to the circulating levels. The proadipogenic role of leptin may be of especial relevance in short/medium term in lean animals under conditions of positive energy balance since it may contribute to the increase in the number of fat cells and hence to the enlargement in the size of fat depots. However, given the effect mentioned above of leptin at high concentrations, chronic hyperleptinemia generally present in obesity could lead to the inhibition of preadipocyte proliferation, but this action could be irrelevant in the leptin-resistant situation generally associated with the obese state [107].

### Apoptosis

Many studies in rodent models have shown a reduction of white fat pads in response to central leptin administration, which has been associated with their effects influencing lipid metabolism [89–91]. Notably, some studies have indicated that the leptin-induced fat depletion may also be achieved by their intriguing effects inducing WAT apoptosis, at least in some fat depots [109–111]. Central leptin administration in rats was shown to entail a reduction in the size of different white fat pads, but only the inguinal depot underwent a significant decrease in cell number and exhibited apoptosis, with positive regulation of the pro-apoptosis Bax protein and increased DNA fragmentation and DNA laddering [110]. Subcutaneous leptin treatment was also shown to induce apoptosis in leptin-deficient (ob/ob) mice in different fat depots (retroperitoneal, inguinal and parametrial), but only in the retroperitoneal depot in the case of ob/? mice, suggesting that ob/ob mice are more sensitive to the leptin action on apoptosis, in addition to differences in the responsiveness among fat depots [112]. Based on findings showing that apoptosis may be induced by activation of βAR in mice [113], it has been proposed that the SNS also mediates the effects of leptin on activation of apoptosis, although other factors and mechanisms may also be involved [112].

Besides central actions, leptin may also exert peripheral effects activating apoptosis, but the mechanisms involved have not been clearly established. In this regard, peripheral administration of leptin (0.1–5 mg/g, intraperitoneal) in C57Bl-ob/ob mice was shown to result in tissue-specific induction of angiopoietin-2 (ANG-2, *Angpt2* gene), with no parallel induction in the vascular endothelial growth factor (VEGF), providing a strong angiostatic rather than angiogenic signal in WAT, and this coincided with the initiation of apoptosis in adipose endothelial cells [114]. The authors also showed that leptin treatment to cultured adipocytes (3T3-F442A cells) also induced *Angpt2* gene expression, providing evidence of a direct autocrine action of leptin in adipose tissue [114].

Therefore, induction of WAT apoptosis may also contribute to the effects of leptin on the reduction of fat depots, mainly acting at the central level, although mechanisms involved need to be further explored. Unlike WAT, no effects of leptin inducing BAT apoptosis have been described [110].

### Thermogenesis and browning

**Fig. 2:**
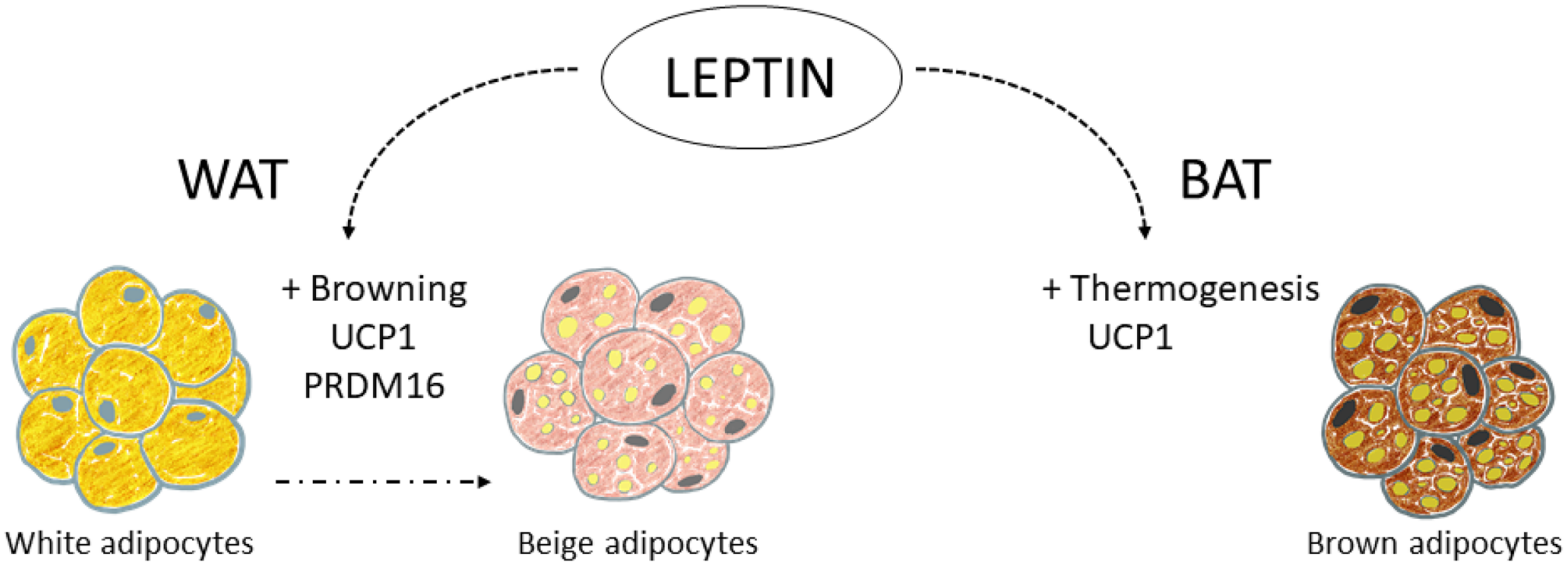
Leptin action on thermogenesis and browning in the adipose tissues. Leptin has been described to activate thermogenesis in brown adipose tissue (BAT), increasing UCP1 (uncoupling protein 1) production. It also exerts browning stimulation in white adipose tissue (WAT), leading to the formation of beige adipocytes, with a greater number of mitochondria, and expressing UCP1 and other markers of browning, such as PRDM16 (PR/SET domain 16). Leptin seems to exert these processes mainly acting on specific leptin-responsive neurons of the central nervous system that regulate sympathetic nervous system outflow to BAT and WAT, but direct/autocrine effects of leptin may also be involved

Non-shivering thermogenesis is the process of heat production due to metabolic processes located primarily in BAT and controlled by the activity of the SNS, not associated with the muscle activity of shivering [115]. The molecular basis of BAT thermogenesis is given by the functioning of UCP1, an inner-membrane mitochondrial protein that uncouples the oxidative respiration from the synthesis of ATP, resulting in energy release as heat [25]. Norepinephrine (NE), released by the SNS in response to stimulus, such as cold and chronic overeating, is the main positive regulator of both UCP1 synthesis and activity, interacting with β3AR on BAT [25, 26]. BAT was traditionally studied and considered important in small mammals (as rodents), but the discovery of the presence of metabolically active BAT in adult humans [116], together with *in vivo* transdifferentiation of white adipocytes into beige or “brite” (brown in white) adipocytes (browning process) [117–119], rekindled their interest as potential therapeutic targets in humans. Leptin is involved in both thermogenesis induction and browning, at different levels (Fig. 2).

Direct evidence of the stimulation of BAT thermogenesis by leptin was established years ago in rodent models. Central leptin administration was shown to stimulate the expression of *Ucp1* in BAT tissue of rats, through β_3_ARs and dependent on sympathetic activation [120, 121]. Other studies have also shown that intracerebroventricular treatment of adult rats with leptin increased mRNA levels of *Ucp1* and *Ucp3* in BAT, and of *Ucp3* in the retroperitoneal and epididymal WAT depots [122]. Subcutaneous injection (20 mg/kg/day) of leptin to lean mice for 1 to 14 days also resulted in gender-dependent complete depletion of fat stores, by day 3 to 4 in females and by day 7 to 8 in males, and this was associated with a rapid, increase in *Ucp1* mRNA levels in BAT [101]. Leptin treatment of ob/ob mice also induced a 4- to fivefold increase in *Ucp1* mRNA expression in BAT and in retroperitoneal WAT, with an equivalent increment of UCP1 protein [123]. Notably, leptin treatment (6 μg/g) was able to increase interscapular BAT temperature in control, diet-induced obese and ob/ob mice, suggesting that thermogenic response to leptin is intact in the obesity models studied in spite of systemic leptin resistance [124]. Other studies in wild type and UCP1-KO mice also evidenced that the reduction of fat depots by leptin treatment was due not only to reduced energy intake but also to increased energy expenditure triggered by UCP1 [125].

Brite adipocytes are inducible thermogenic cells that appear as clusters inside WAT depots, showing a combined energy storage/expenditure function and a mixed multilocular/unilocular/paucilocular morphology [21]. Brite cells share some common features with brown adipocytes, such as great number of mitochondria, expression of UCP1 [126] and other markers of browning, such as PR/SET domain 16 (PRDM16) [21], whereas they differ in their localization, developmental origin, cell size, expression of specific genes, etc. [127]. The main activator of browning and thermogenic activity seems to be the sympathetic induction of WAT [128]. Leptin has the potential of browning induction in WAT. Central leptin administration in mice has been shown to activate WAT browning through the activation of phosphatidylinositol 3-kinase (PI3K) signalling in the CNS, and the subsequent stimulation of the sympathetic nerve activity to the WAT [129]. Notably, co-infusion of leptin and insulin prompted a greater induction of WAT browning, suggesting a synergistic action of both hormones on the POMC neurons of the ARC and connecting the PI3K signalling pathway [130]. Furthermore, the authors also showed attenuation in WAT browning after sympathetic denervation, indicating that the browning process is dependent on the autonomic nervous system [130]. Similarly to the effects described in BAT, subcutaneous leptin administration in mice has also been described to activate WAT browning [101], and adenovirus-induced chronic hyperleptinemia in lean Zucker rats has also been associated with a transformation of WAT, characterized by depletion of adipocyte fat, as well as deep down-regulation of genes related to lipogenesis and upregulation of genes associated with FA oxidation and uncoupling proteins 1 and 2 [103], in agreement with a browning induction by leptin.

### Inflammation

Leptin is a key modulator of the immune system, with a wide range of functions both in innate and adaptive immune responses [131]. In general, leptin exerts proinflammatory properties, upregulating the secretion of proinflammatory cytokines, such as tumour necrosis factor (TNF)-α, interleukin (IL)-6, and IL12 [132, 133]. In addition, most of the cells of the immune system express and present the LEPR in their membranes, and leptin has been shown to activate immune cells both recruited and resident in the adipose tissue, including macrophages, inducing the production of cytokines implicated in the inflammatory process [134, 135]. It is remarkable that macrophage infiltration in adipose tissue is increased in obesity, participating in the inflammatory pathways that are activated under these conditions [136, 137]. Therefore, high leptin concentration may promote inflammation of adipose tissue, and the chronic hyperleptinemia characteristic of obese patients has been proposed to contribute to the low-grade inflammatory state that characterizes obesity and makes subjects more susceptible to developing type II diabetes, cardiovascular disease, as well as other pathologies, such as autoimmune diseases [133].

Nevertheless, the effects of leptin on adipose tissue inflammation may depend on leptin concentrations and animal conditions. Leptin treatment within a subphysiological to physiological range (0.1, 0.5, and 3.0 mg/kg/day) to leptin-deficient low-density lipoprotein receptor (LDLR) knockout (LDLR-/-;ob/ob) female mice for 12 weeks resulted in reduced macrophage infiltration in the epididymal WAT, lower mRNA levels of the pro-inflammatory IL6 and MCP1 (monocyte chemoattractant protein 1) proteins in different WAT depots and interscapular BAT, and diminished IL6 and MCP1 plasma levels [138]. In line, leptin treatment in ob/ob mice was shown to trigger catecholamine-dependent increases in cAMP signalling that reduced inflammatory gene expression in adipose-resident macrophages through activation of the histone deacetylase HDAC4 [139]. Moreover, macrophage infiltration in WAT and mRNA levels of the inflammation markers MCP1, F4/80, and TNFα were found to be diminished in two mouse models of partial leptin deficiency (OBHZ and LepHZ mice), after a chronic high-fat (HF) diet feeding, suggesting that lower leptin levels within an obesogenic environment may be beneficial for obesity and insulin development prevention [140].

## Long-lasting effects of leptin during the perinatal period on adipose tissue programming, growth and development

There is compelling evidence of the link between adverse perinatal environment and increased risk of obesity and metabolic-related disorders in later life, conceptualized in the 1990s as the “developmental origins of health and disease” hypothesis [141]. More recent studies have shown that factors or conditions during a critical window of developmental plasticity, particularly gestation and lactation, have the capacity to programme the structure and function of adipose tissue, besides other key organs and tissues, with implications in body weight control and obesity risk [142]. A variety of perinatal insults, including undernutrition during gestation [143–145] and maternal obesity/obesogenic diet [146] have been shown to have a big impact on adipose tissue development, programming offspring for increased adiposity. Leptin, along with other hormones, such as insulin and steroid hormones, which play essential roles in cell proliferation, apoptosis, neurodevelopment, among other physiological functions, appear as potential targets of perinatal insults, and alterations in their concentrations or function may have negative impact on metabolic programming [147–150].

### Programming effects of leptin during gestation

The potential relation between leptin levels during gestation and the programming of adiposity has been studied by analysing leptin levels in different extraembryonic/foetal compartments, including maternal blood, placenta, umbilical cord, and foetal circulation, with divergent associations. Studies have been carried out in models such as rodents, ewes and humans, where the various perinatal timing of development of adipose depots is essential. While adipose tissue is mainly developed between late gestation and the first four postnatal weeks in rodents [151, 152], in humans, the first appearance of adipose tissue depots starts early, between weeks 14–24 of gestation (i.e. at the beginning of the second trimester) [152]. Therefore, the influence of hormonal and environmental signals on adipose tissue development during gestation in humans might be highly relevant in the metabolic programming and development of offspring adipose tissue.

The research so far regarding the relationship between maternal circulating leptin levels during pregnancy and adiposity in the offspring has given some varied or inconclusive results, with a need for more longitudinal research. In humans, maternal circulating levels of leptin increase in the first and second trimester (peaking at about week 28), explained both by an increase in maternal adipose tissue and placental production (in fact, there is 95–98% release of placental leptin to maternal circulation, and in lower quantity to foetal circulation); moreover, the levels of the soluble leptin receptor (LEPRe) also raise, helping to maintain elevated circulating leptin levels [148, 153]. The association of increased adipose tissue mass and adipocyte hypertrophy in the offspring with maternal obesity (associated with elevated blood levels of leptin and insulin, which may enhance lipo- and adipogenesis) has been suggested, and the link between maternal BMI and increased risk of comorbidities in the offspring seems clear (given by meta-analyses and systematic reviews) [152, 153]. Nevertheless, the results concerning maternal (circulating) leptin levels as a putative predictor of birth weight or body fat or obesity risk in the offspring are inconclusive or even divergent when comparing different studies with neonates or which follow cohorts of children until 1 to 7 years (e.g. some recent studies: [154–157]. In one of the studies [156], the authors found a positive association of skinfold thickness (as an indicator of adiposity in neonates) with free leptin (calculated as plasma leptin/soluble leptin receptor) in women with pregestational obesity but negative in non-obese mothers.

During pregnancy, the placenta is an important place for leptin expression and production (mainly in the syncytiotrophoblast and villous vascular endothelial cells), also expressing the leptin receptors, having autocrine, paracrine and endocrine effects. Moreover, a specific upstream enhancer works in the placental tissue, but not in adipose cells, explicitly mediating placental leptin expression [152, 153, 158], therefore highlighting the key role of placental leptin during pregnancy. Leptin and its receptor have different physiological roles during gestation, such as critical roles in implantation, placental growth and function, and maintenance of pregnancy [152]. The expression of leptin and its receptor is increased in the placentas from women with gestational diabetes, activating protein synthesis and therefore partly explaining the increased foetal growth associated with gestational diabetes [153]. Leptin also activates amino acid transport capacity in the placenta, which may be a determinant factor in the foetal overgrowth associated with maternal obesity [152].

Differently to maternal levels, leptin concentration in umbilical cord blood is generally accepted as a marker of adiposity in neonates and has been shown to positively correlate with birth weight, to influence infant growth trajectories, and to inversely correlate to BMI in early infancy [159–162]. Body weight is not a good marker of adipose accretion and, instead, the direct study of adiposity is more relevant but less investigated. In this sense, Chaoimh et al. [163] have shown, also in early infancy (2 months), that an inverse association of cord blood leptin levels with conditional weight gain is mainly driven by a lower increase in adiposity. Other results have shown an inverse association between cord blood leptin and adiposity at 3 years [164]. But these effects might not be consistently shown at different ages. For instance, Meyer et al. [154] did not find sufficient evidence (with a relatively small cohort) that leptin levels in cord blood might be a predictor of fat distribution or obesity in early childhood (from 3 to 5 years), while Simpson et al. [165] have reported that cord blood leptin can weakly be associated to increased fat mass in late childhood (9 years). Therefore, the value of cord blood leptin concentration as a predictive marker of fat accretion throughout infancy deserves more research. Moreover, gender differences in cord leptin levels and the degree of association with growth have been found (e.g. [155, 162]), therefore sex-specific effects also deserve more-in-depth study.

During gestation, leptin produced by the foetus may also be highly relevant in metabolic programming of adipose tissue, both directly or through brain (especially hypothalamic) circuit developmental regulation. The effects of leptin on foetal growth and development may be tissue-specific; e.g. it has been shown to affect the development and migration of neuronal and glial cells in the murine foetal brain [148, 166]. Moreover, foetal circulating leptin and foetal adipose mass are related, suggesting that the function as an adipose reserve signal of leptin is similar as in postnatal life [148]. As mentioned above, leptin has a critical role in the formation of the hypothalamic neural circuits regulating food intake, and there are beneficial effects of leptin supplementation during lactation in rodents (see next section), coinciding with the main development of the hypothalamic appetite regulatory network (it occurs predominantly after birth in rodents). In the case of more precocial species, such as humans and sheep, there is a prenatal development of the appetite regulatory neural network and important deposition of fat before birth [167]; therefore, an important role of foetal leptin in energy balance metabolic programming, and the consequent effect on adipose tissue development, might be expected. Considering that leptin synthesis and circulating levels in the foetus are regulated or altered by a variety of intrauterine and environmental conditions and that it might signal energy/nutrient availability, especially during late gestation [148], the study of this regulation, the environmental factors involved, and the long-lasting consequences in later life are relevant.

**Fig. 3:**
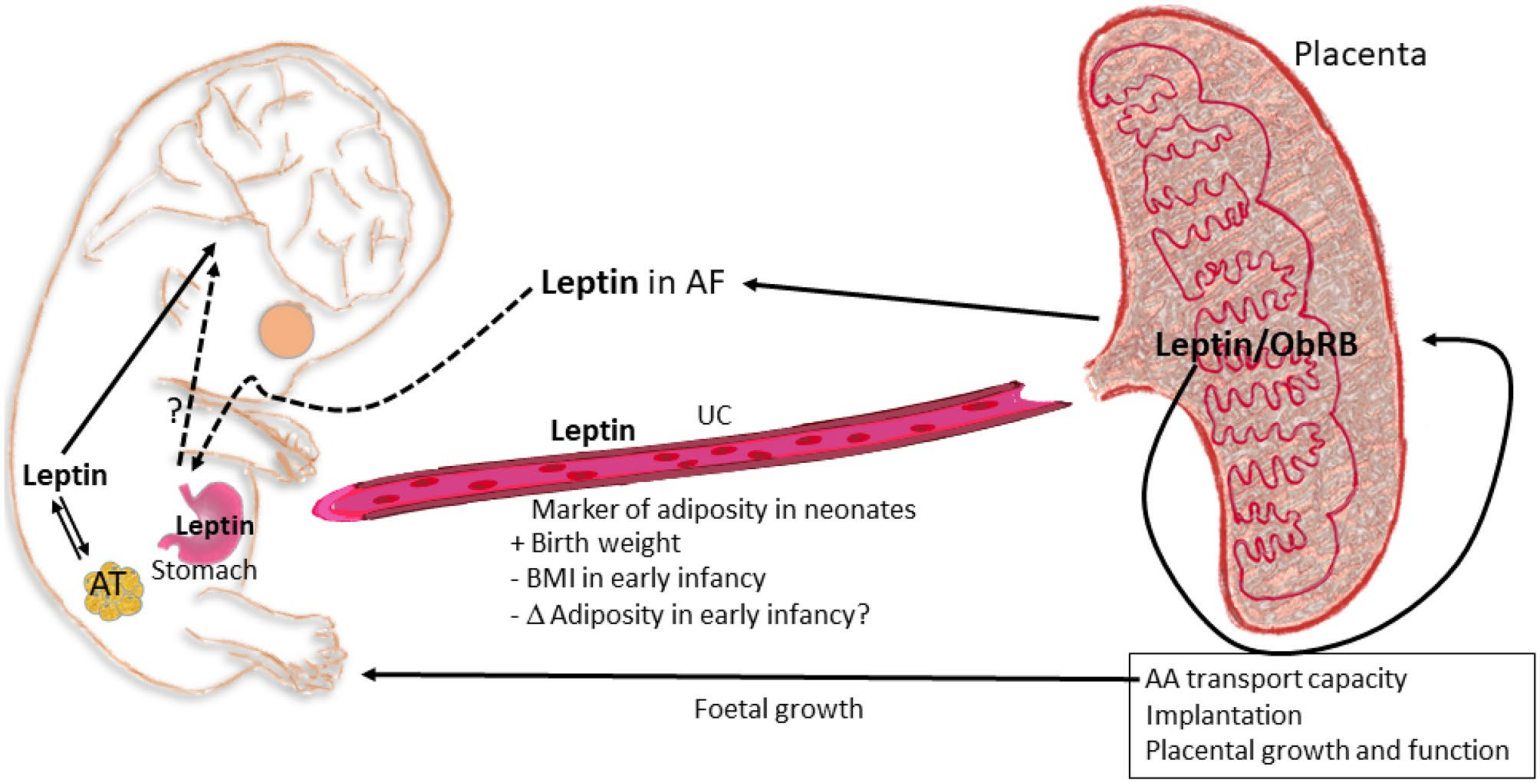
Putative roles of embryonic and extraembryonic leptin on foetal and adipose tissue development. The placenta expresses both leptin and its full signalling capacity receptor (LEPRb), regulating critical functions of placenta during pregnancy, including amino acid (AA) transport capacity, related to foetal growth. Present evidence suggests that leptin levels in umbilical cord (UC) are a marker of adiposity in the neonates and may be putatively related to less adiposity accretion, at least in early infancy. Leptin produced by the foetus has been suggested to be related to the regulation of metabolic programming through brain (particularly hypothalamus) and adipose tissue (AT). More recently, preliminary evidence has pointed out the possibility (in a rodent model) that leptin in amniotic fluid (AF) in late gestation might have a putative physiological role in near-term foetuses, trough stomach leptin internalization, after swallowing

By the evidence discussed above, it is clear that much is pending to investigate regarding leptin in different foetal/extraembryonic compartments, its regulation, and the effects on metabolic programming and adiposity in later life. We have recently considered the possible physiological function in late gestation of leptin in amniotic fluid, its relation with placental leptin, and its potential role as a putative signal internalised by the foetal stomach by amniotic fluid swallowing in a rodent model [168]. In brief, leptin in quantifiable levels shows a sudden appearance in amniotic fluid at day 20 of gestation (near term, in rodents), which might be related to changes in placental leptin localisation and can be internalised by the immature stomach, with a potential physiological role in near term foetuses. The potential roles of embryonic and extraembryonic leptin on foetal and adipose tissue development are summarised in Fig. 3.

### Programming effects of leptin during lactation

There is clear epidemiological evidence of the benefits of breastfeeding, compared with formula feeding, on the offspring health later in life. Breastfeeding confers short term beneficial effects in infants, protecting against the incidence of infections, asthma, allergies, etc. [169], but also confers protection against later obesity and diabetes, and has modest effects on risk factors of cardiovascular diseases [170, 171]. Benefits of breastfeeding have been primarily attributed to differences in the caloric content and macronutrient composition of breastmilk in comparison to infant formula, but may also rely on the presence of bioactive factors that are not present, or not at the same concentration, in commercially available infant formula [172]. Among these differential factors, breast milk contains a myriad of endogenously synthetized peptide hormones and cytokines, some of them with a relevant function in energy metabolism (e.g. leptin, insulin, adiponectin, ghrelin, resistin, etc.), in addition to other compounds (such as miRNA) [18, 173, 174]. Among them, probably the most biologically relevant, and for which there is compelling evidence of its physiological importance during lactation in metabolic programming, is leptin, as can be deduced from our intervention studies in rodents [175–178] and indirect epidemiological studies in humans [18].

Leptin is produced by the mammary epithelium [16], and is present in breast milk [179, 180], but not in infant formula [181]. Leptin present in milk comes from its production in the epithelial cells of the mammary gland and from maternal circulation [180]. Breast milk leptin levels vary depending on maternal adiposity. A positive correlation has been consistently reported in most studies between maternal BMI/adiposity and breast milk leptin concentration, as well as between leptin levels in maternal blood and breast milk, so that mothers with obesity have greater amounts of leptin in breast milk than normal-weight mothers [18, 182]. Notably, we first described in a group of 28 non-obese women the existence of a negative correlation between leptin concentration in breast milk and both BMI and body weight gain of their infants until to two years of age [175, 182]. Most of the subsequent studies performed in infants by independent groups confirmed our previous findings, although some studies found no clear correlation (reviewed in [18]). Differences between studies may be attributed to the bias due to the inclusion of women with obesity, as it appears that obesity impairs the function of leptin in breast milk [18] by mechanisms that are under investigation. It could be speculated that excessive intake of leptin during lactation might not provide greater protection against excess weight gain or could even favour the development of leptin resistance later in life, although these possibilities have not been specifically addressed. It would be interesting to further explore the relationship between leptin levels in breast milk and body weight/adiposity and related disorders in human adult offspring.

Animal studies have provided direct demonstration of the essential role of leptin ingested during the suckling period for the proper development of the neonate [175, 176]. Male rats that were supplemented with physiological doses of leptin during the suckling period were more resistant to overweight/obesity and related metabolic complications both under standard diet and when exposed to a HF diet [176, 178]. They were also more protected against the age-related increase in body weight/adiposity [177]. The effects of leptin were first attributed to increased central leptin sensitivity and related to changes in the hypothalamic expression of genes involved in leptin action [176]. Specifically, animals that were supplemented with leptin during the suckling period displayed lower mRNA expression of the gene encoding for the SOCS-3 protein, which inhibits leptin signal transduction. In addition, they maintained “normal” *Lepr* expression levels under HF diet conditions, which contrasted with the lowered expression in rats that did not receive such supplementation [176]. Leptin also ameliorated the lasting detrimental effects of a HF diet on the peripheral leptin action, evidenced by the maintenance of LEPR abundance in WAT, and associated with increased oxidative capacity in this tissue [178]. Animals that were supplemented with leptin during the suckling period also showed improved insulin sensitivity [177] and were protected from other diet-induced metabolic disturbances, such as hepatic lipid accumulation [178]. Therefore, leptin play key biological actions during the perinatal period with long-lasting outcomes in the maintenance of energy homeostasis.

What are the specific actions of leptin during breastfeeding? Studies carried out in animal models have allowed finding out that leptin has a critical neurotrophic action during the suckling period and is necessary for the proper development of hypothalamic circuits that are involved in body weight control [18, 72, 147]. In rodents, this action is restricted to a critical period during lactation, around the second postnatal week, coinciding with a transient increase in circulating levels of leptin, the so-called leptin surge [147]. Lack of leptin during this period, as occurs in leptin-deficient mice [147], or alterations in leptin surge due, for example, to poor perinatal conditions (e.g. gestational mild caloric restriction) [75, 76], compromise the neuronal organization of the hypothalamic nuclei involved in the food intake control as well as other regulatory centres, thereby impairing the ability to regulate energy homeostasis in adulthood. Interestingly, exogenous leptin treatment during the suckling period, but not in adulthood, has been shown to promote neuronal development and allow to recover the arcuate nucleus neural projections that are disrupted in genetically deficient mice, indicative of the essential role of leptin during this period [147]. Likewise, oral supplementation with physiological doses of leptin throughout lactation was shown to ameliorate alterations in the CNS structures, particularly in hypothalamic arcuate nucleus structure and function due to moderate gestational calorie restriction [183], a condition associated with the disruption in the leptin surge during the postnatal period [76]. The action of leptin during lactation did indeed translate into a healthier phenotype in adulthood, particularly evident in rats exposed to an obesogenic diet [184]. In these animals, leptin supplementation largely prevented the dysmetabolic phenotype associated to undernutrition during gestation, characterized by increased fat accumulation and other metabolic syndrome-related disturbances, such as insulin resistance, hypertriglyceridemia, and hepatic steatosis [184]. This represents a demonstration that the intake of physiological doses of leptin during lactation may reverse neuroanatomical defects and the programmed trend for obesity and related risk factors acquired by adverse conditions during pregnancy, which is of great interest as a strategy to treat or prevent the development of obesity. The neurotrophic action of leptin during early stages of development precedes their ‘classic’ function in body weight control in later stages [185]. Nevertheless, having received leptin during this critical period of development seems crucial for leptin to exert its effects adequately in later stages, at least concerning the control of energy homeostasis.

In addition to the effects of leptin on the development of CNS structures involved in body weight control, leptin may exert programming effects on adipose tissue development and function [119]. Some clues have been obtained from studies in animal models exposed to perinatal nutritional disturbances. Gestational undernutrition in rodent models has been shown to affect the development of peripheral nervous system structures, including sympathetic innervation of white [143] and brown [144] adipose tissues. Reduced sympathetic innervation markers in inguinal WAT were described in the male offspring of calorie-restricted rats during gestation, associated with a reduced oxidative capacity of their adipocytes, and accompanied by hyperplasia and increased fat accumulation in adulthood [143]. Notably, leptin supplementation at physiological doses throughout lactation restored WAT sympathetic innervation and normalised phenotypic expression of genes related to lipolysis (*Pnpla2*, *Lipe*) and FA oxidation (*Cpt1b*, *Ppargc1a*) in this fat depot in weaned rats [186]. It is expected that these changes also contribute to preventing the programmed predisposition for increased fat accumulation and other metabolic disturbances in adulthood, as demonstrated in further studies [184].

Lipogenesis and lipolysis in WAT are also regulated by thyroid hormones, in addition to the action of the SNS [187]. The normalisation of WAT function in the offspring of calorie-restricted rats during gestation by leptin supplementation during the suckling period was also shown to involve normalisation of decreased plasma T3 levels and altered signalling in WAT [186]. This action may also account for the normalization of lipoprotein lipase (*Lpl*) mRNA levels and the impaired capacity to mobilize fat in these animals, particularly males [186].

**Fig. 4:**
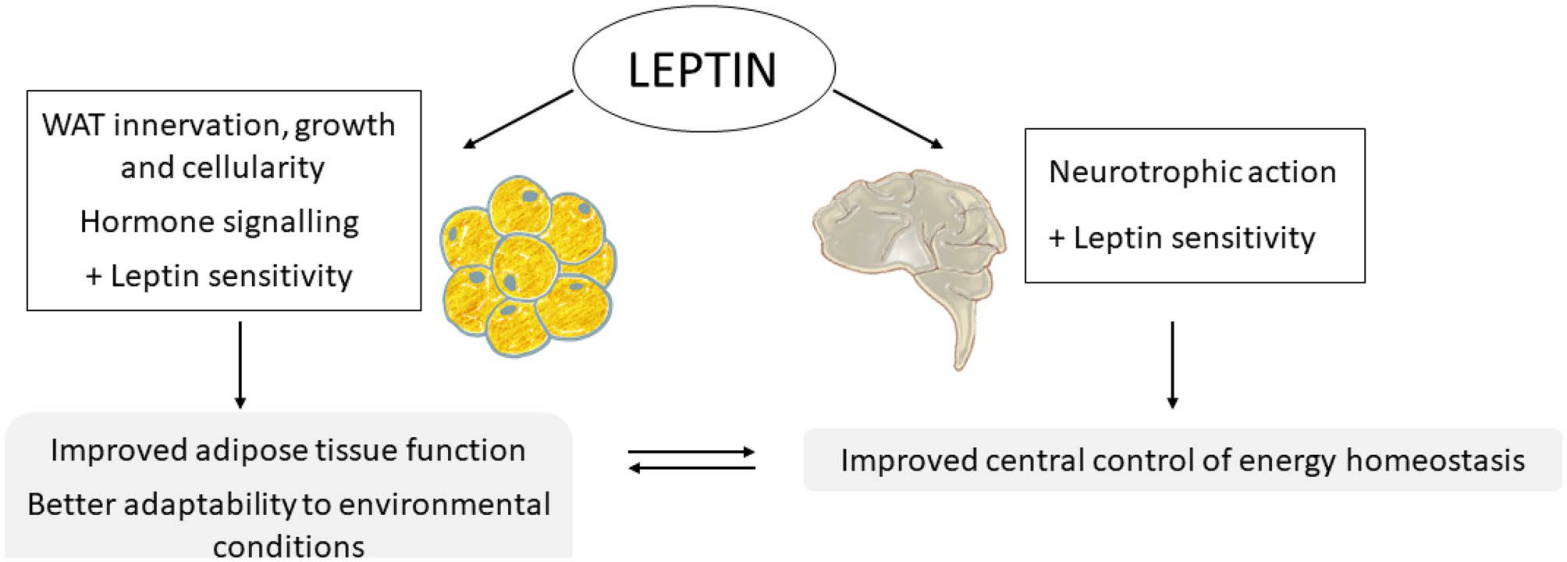
Programming effects of leptin during lactation on white adipose tissue (WAT) and brain. Leptin intake during lactation has been described to exert neurotrophic actions, and is necessary for the proper development of hypothalamic circuits that are involved in body weight control. Leptin action during lactation is also associated with increased central leptin sensitivity, which improves the later control of energy balance. Moreover, leptin during the suckling period regulates innervation, growth and cellularity of WAT, and improves hormone signalling in this tissue, including that of leptin itself. Therefore, the intake of adequate doses of leptin during the suckling period leads to an improvement in the characteristics and functionality of the adipose tissue, programming it for a greater capacity to adapt and respond to different environmental conditions. These effects, together with those it exerts on the development of the hypothalamus, as well as on other tissues, result in a better ability to control energy homeostasis and prevent metabolic syndrome-related alterations

In short, there is still much to explore about the role of leptin during early stages of life on the development and function of adipose tissue. However, the fact that central and peripheral leptin signalling seem to be blunted in animal models exposed by maternal malnutrition during pregnancy and improved by leptin supplementation during the lactation period, suggests that leptin may exert an essential role, not only in the proper development of hypothalamic circuits involved in the control of energy balance, but also in metabolic programming of adipose tissue and its capacity to respond to leptin and to adapt to different environmental conditions (Fig. 4).

## Concluding remarks

Since leptin was discovered in 1994, it continues to amaze us the variety of functions that this hormone can exert beyond the initially envisaged in body weight control, the targets on which it acts, as well as the different sources of this hormone.

**Fig. 5:**
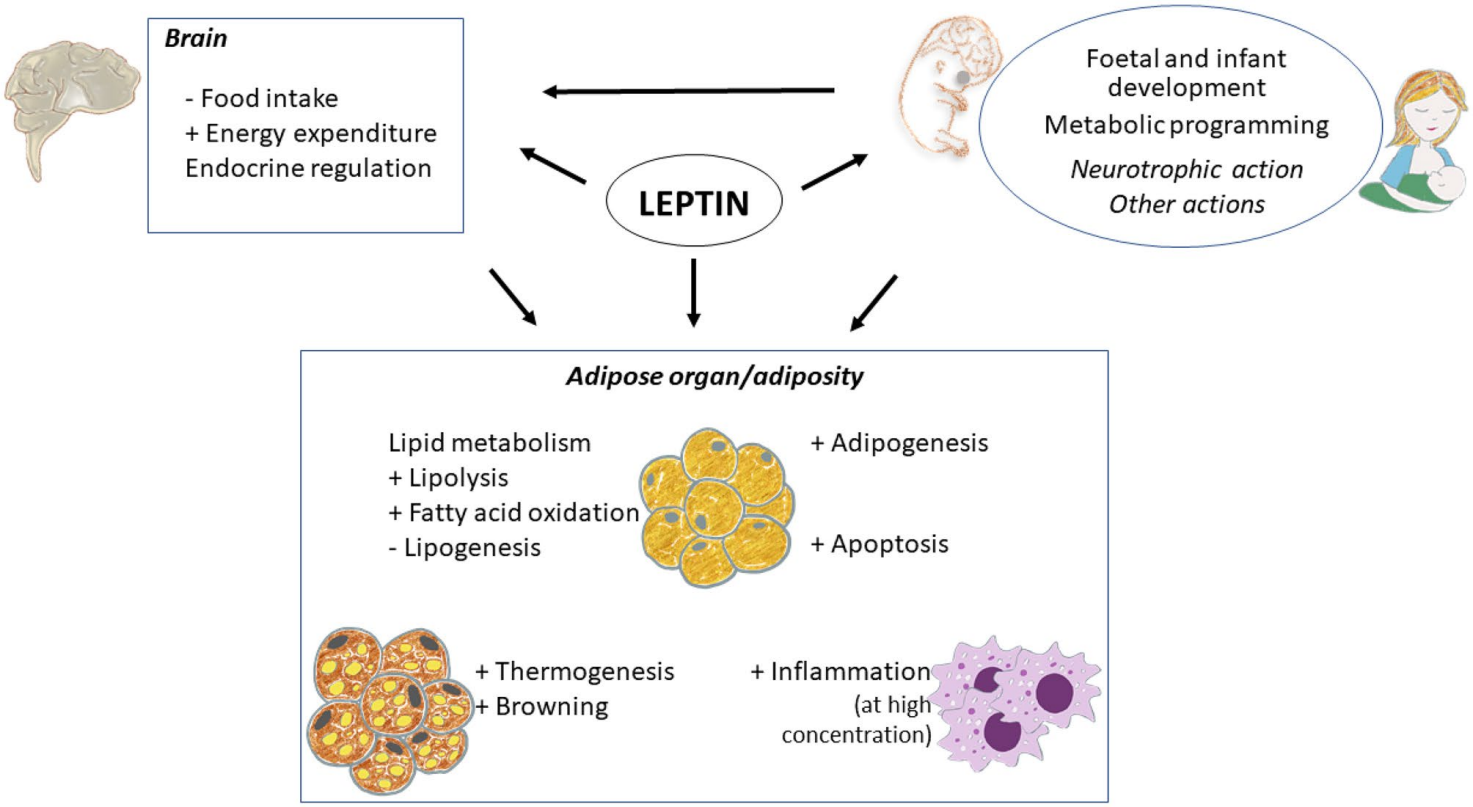
Overview of leptin actions in the adipose organ. Leptin exerts central actions, modulating energy homeostasis and influencing multiple endocrine actions, and peripheral actions in different tissues. In the adipose organ, leptin exerts key regulatory actions through direct or indirect mechanisms, with short/medium- and long-term implications. Leptin promotes adipogenesis, lipolysis, fatty acid oxidation, apoptosis, browning, and stimulates BAT thermogenesis. At high concentrations, leptin also stimulates the production of pro-inflammatory cytokines and promotes inflammation. Moreover, during critical windows of development, leptin may exert central neurotrophic actions, affecting leptin-sensitive brain circuits involved in adipose tissue regulation, which can lead to morphological and physiological changes in the adipose tissue, with long-lasting consequences for later function and metabolic health. Therefore, adequate amounts of leptin during the perinatal period, both during the foetal and suckling periods, seem to be essential for the proper development of the adipose organ and may also compromise later leptin action. A better understanding of the mechanisms and actions of leptin in the adipose organ, as well as in other target tissues, may lead to new approaches in obesity prevention and treatment since the early stages of life

Adipose tissue is one of the main targets of leptin, and its action is key in the control of energy metabolism, since it regulates critical functions, such as lipid mobilization and oxidation, lipid synthesis, or such essential processes that determine the size of fat reserves, as adipogenesis and apoptosis, as well as energy expenditure-related processes (thermogenesis in brown adipose tissue and browning of white adipocytes), among others. Moreover, the function of leptin is important throughout life. A new role of leptin in metabolic programming has been recently unveiled, pointing to this hormone as an essential component of breast milk and, probably of the amniotic liquid. Leptin is essential during critical windows of development with heightened plasticity. Thus, having adequate leptin levels in extraembryonic/foetal compartments during the foetal stage, and ingesting appropriate amounts of leptin from maternal milk during lactation, appears critical for the correct programming and development of the adipose tissue and structures controlling it and involved in energy metabolism, with long-lasting effects in metabolic health, determining for example later sensitivity to this hormone (Fig. 5). Further knowledge of the role of leptin in adipose tissue, mechanisms and pathways involved, and its regulation may be of interest in the design of new strategies for the prevention and treatment of obesity and related pathologies, even from early stages of life.
